# Supplementary material for: RILP Induces Cholesterol Accumulation in Lysosomes by Inhibiting Endoplasmic Reticulum–Endolysosome Interactions
Source: Cells. 2024 Aug 6;13(16):1313. doi: 10.3390/cells13161313 (PMC11352460; doi:10.3390/cells13161313)
Supplement: Supplementary file 1 [file cells-13-01313-s001.zip › Supplemental figure legends.pdf]

Supplemental figure legends

Figure S1 (A) Hela cells were transfected with GFP-ORP1L, and immuno-labeled with Lamp1, showing dispersed distribution of ORP1L. (B) Hela cells were effectively infected with lentivirus expressing PCDH-CMV-Cherry-RILP to achieve all cells expressing RILP. (C) Western blot detected the lentivirus-mediated expression of RILP. (D) Western-blot detected the Lentivirus-mediated gene silence by shRNA-RILP, and shRNA-RILP-2 was used in this study. (E) Western-blot detected the Lentivirus-mediated gene silence by shRNA-Rab7, shRNA-Rab7-2 and shRNA-Rab7-3 was used in this study.

Figure S2 (A) Hela cells were transfected with GFP-RILP and then the expression of Rab7 was knocked down by shRNA-Rab7, and then immunostained with antibody against Lamp1. Immunofluorescence microscopy to reveal that RILP still results in clustering of the late endosomes/lysosomes. (B) Hela cells were infected with RILP knockdown lentivirus and labeled with Filipin to detect cholesterol distribution. Immunofluorescence microscopy revealed that RILP knockdown resulted in peripheral distribution of cholesterol. (C) Hela cells labeled with anti-Lamp1 were cultured in DMEM supplemented with cholesterol or U18666A, respectively. Immunofluorescence microscopy revealed that high cholesterol treatment induces peri-nuclear distribution of the late endosomes/lysosomes. Bar=20μm.

Figure S3 (A) Hela cells were treated with different concentrations of Torin1 (which induces autophagy). The protein level of RILP was detected by western blot. The results showed the expression of RILP increased in a dose dependent manner. (B) PCR approach was used to detect the mRNA levels of RILP. The results showed the transcription of RILP is upregulated in high fat-fed diabetes type 2 mice. (C) and (D) Hela cells were infected with lentivirus expressing PCDH-CMV-Cherry-vector or PCDH-CMV-Cherry-RILP. Then, western blot was used to detect p-mTOR, p-ULK1, mTOR, ULK1 and GAPDH. (E) Quantitative analysis of the results of C and D from 3 independent experiments. (F) The representative TEM images of Hela cells infected with PCDH-CMV-Cherry-vector or PCDH-CMV-Cherry-RILP, autophagosomes are marked by a bilayer membrane. (G) and (H) Immunogold staining experiment was carried out to show the location of RILP.

Figure S4 293T cell lysates containing GFP-OPRs were processed for GST-pulldown experiments using GST-RILP. The bond ORP proteins were detected by Western blot using GFP antibody.
