# Supplementary material for: RILP Induces Cholesterol Accumulation in Lysosomes by Inhibiting Endoplasmic Reticulum–Endolysosome Interactions
Source: Cells. 2024 Aug 6;13(16):1313. doi: 10.3390/cells13161313 (PMC11352460; doi:10.3390/cells13161313)
Supplement: Supplementary file 1 [file cells-13-01313-s001.zip › Supplemental Table.pdf]

Supplemental table. Oligos used in the experiments

Primers for generating plasmids

	Forward primer	Reverse primer
GFP-ORP1L	5’ CCGGAATTCTATGAACACAGAAGCAGAACAG 3’	5’ CGCGGATCCTTAATAAATGTCAGGCAAATTAA 3’
GFP-ORP1L(1-468aa)	5’ CCGGAATTCTATGAAGAACAAAAATGATCAGAA 3’	5' CGCGGATCCTTAATAAATGTCAGGCAAATTAA 3'
GFP-ORP1L(1-485aa)	5’ CCGGAATTCCGGTATGACTCACTACTGTTCCCA 3’	5’ CGCGGATCCTTAATAAATGTCAGGCAAATTAA 3’
GFP-ORP1L(486-950aa)	5’ CCAAGCTTGGGCGATGTCCGAAGAAAAAGACTG 3’	5’ GCGGATCCTTAATAAATGTCAGGCAAATTAA 3’
His-ORP1L	5’ CGCGGATCCGCGATGAACACAGAAGCGGAGCA 3’	5’ CCGCTCGAGCGGTTAATAAATGTCAGGCAAA 3’
His-ORP1L(336-950aa)	5’ CGCGGATCCAGCACTCACTACTGTTCCCAGGAC3’	5’ CCGCTCGAGCGGTTAATAAATGTCAGGCAAA 3’
GFP-ORP7	5’ GAAGATCTATGGACTTCCAAGAGAGGGACCC 3’	5’ CCCAAGCTTCTACCAGAGCACGGCCC 3’
GFP-ORP7(1-401aa)	5’ CCGGAATTCTATGGACTTCCAAGAGAGGGACCCG 3’	5’ TCCCCCGGGCTACGTGTGGGAATCAGCAAG 3’
GFP-ORP7(1-409aa)	5’ CCGGAATTCTATGGACTTCCAAGAGAGGGACCCG 3’	5' TCCCCCGGGCTAAACCTCGCAGGCATCGAA 3'
GFP-ORP7(410-842aa)	5’ CCGGAATTCTATGCTCCTCTCCGCCAGCTCTTC 3’	5’ TCCCCCGGGCTACCAGAGCACGGCCC 3’
Cherry-VAPa	5’ CGGAATTCTATGGCGTCCGCCTCAGGGGC 3’	5’ CGCGGATCCCTACAAGATGAATTTCCCTAG 3’
GST-VAPa(1-225aa)	5’ CGGAATTCATGGCGTCCGCCTCAGGGG 3’	5’ CCGCTCGAGCTAGGTGACATTATCTCTGAAG 3’
GST-Rab7	5’ CCGGAATTCATGACCTCTAGGAAGAAAGTG 3’	5’ CCGCTCGAGTCAGCAACTGCAGCTTTCTG 3’
Cherry-RILP	5’ ACGCGTCGACTCAGGCCTCTGGGGCGGC 3’	5’ CCGGAATTCTATGGAGCCCAGGAGGGCG 3’
His-RILP	5’ CCGGAATTCATGGAGCCCAGGAGGGCGGC 3’	5’ CCCAAGCTTTCAGGCCTCTGGGGCGGCTG 3’
RILP-RT-PCR(Rat)	5’ TCAAGGAGGTGACAGACAGACAGA 3’	5’ GAGCATCTCTCGCTGGAAATA 3’
GAPDH-RT-PCR(Rat)	5’ GGAGAAACCTGCCAAGTATGA 3’	5’ TTGAAGTCACAGGAGACAACC 3’

Targeting sequences for shRNA

shRILP-1#	5' CGGAATGAACTCAAAGCCAAA 3'
shRILP-1#	5' CGGAATGAACTCAAAGCCAAA 3'
shRILP-2#	5' GAAGATCAAGGCCAAGATGTT 3'
shRILP-3#	5' GAGTTTCTTTGGCCTATGGTA 3'
shRILP-4#	5' GCGGAGCTTGTGTACCATCTA 3'
shRab7-1#	5' GCCACAATAGGAGCTGACTTT 3'
shRab7-2#	5' GAAACAAGATTGACCTCGAAA 3'
shRab7-3#	5' GGCTAGTCACAATGCAGATAT 3'
