# Supplementary figures and images for: RILP Induces Cholesterol Accumulation in Lysosomes by Inhibiting Endoplasmic Reticulum–Endolysosome Interactions
Source: Cells. 2024 Aug 6;13(16):1313. doi: 10.3390/cells13161313 (PMC11352460; doi:10.3390/cells13161313)

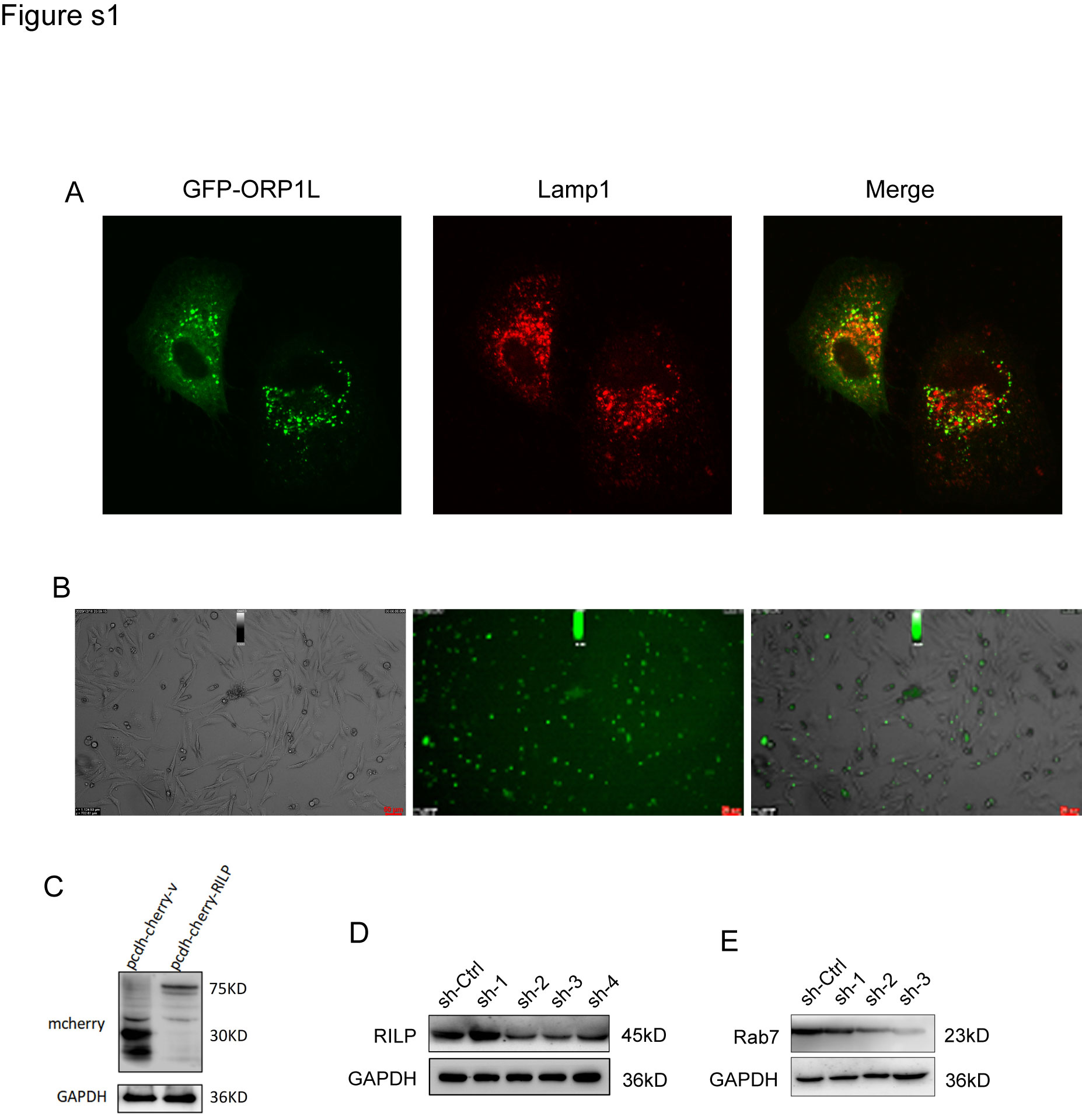

Supplement: Supplementary file 1 [file cells-13-01313-s001.zip › Figure_s1.jpg]

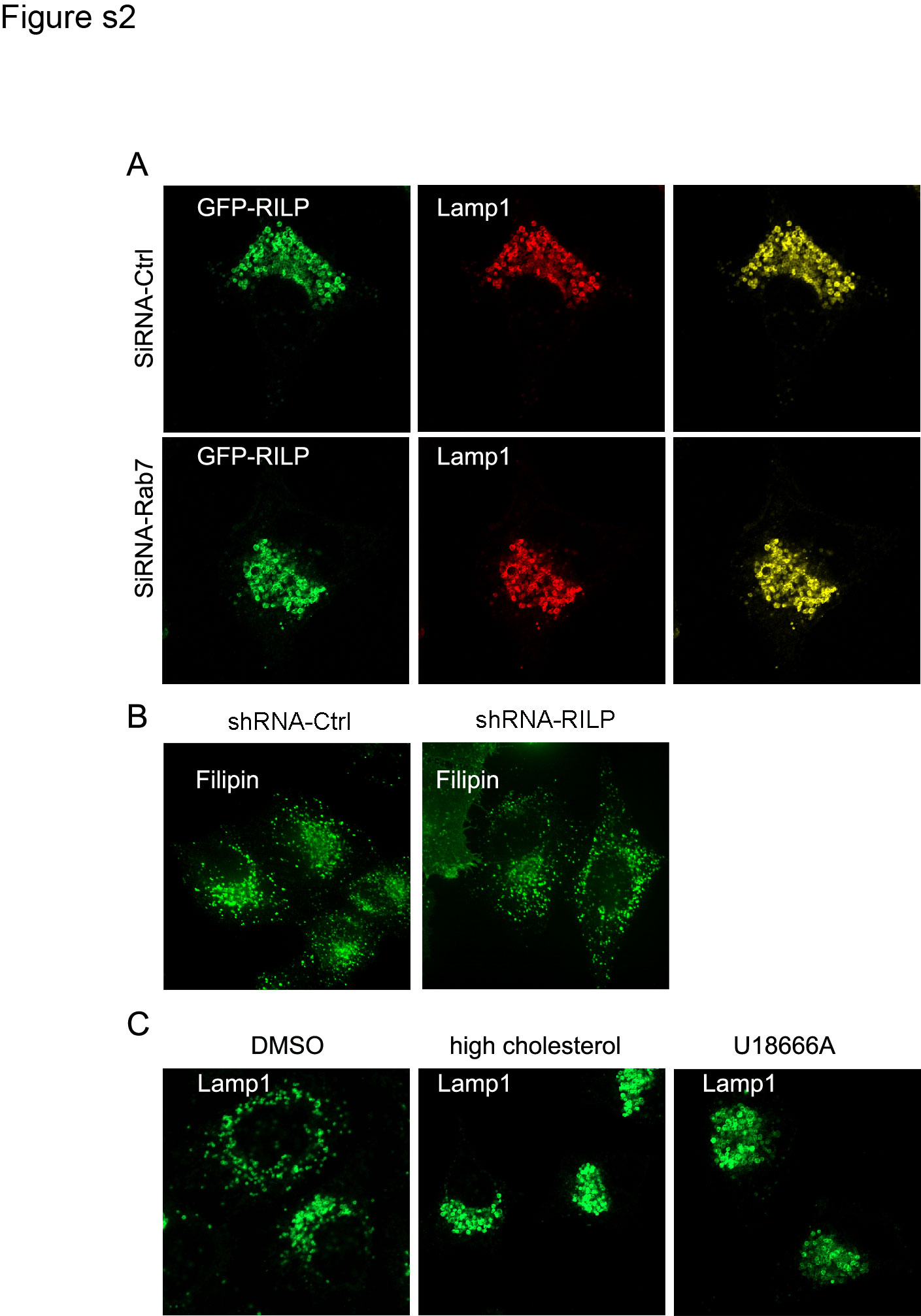

Supplement: Supplementary file 1 [file cells-13-01313-s001.zip › Figure_s2.jpg]

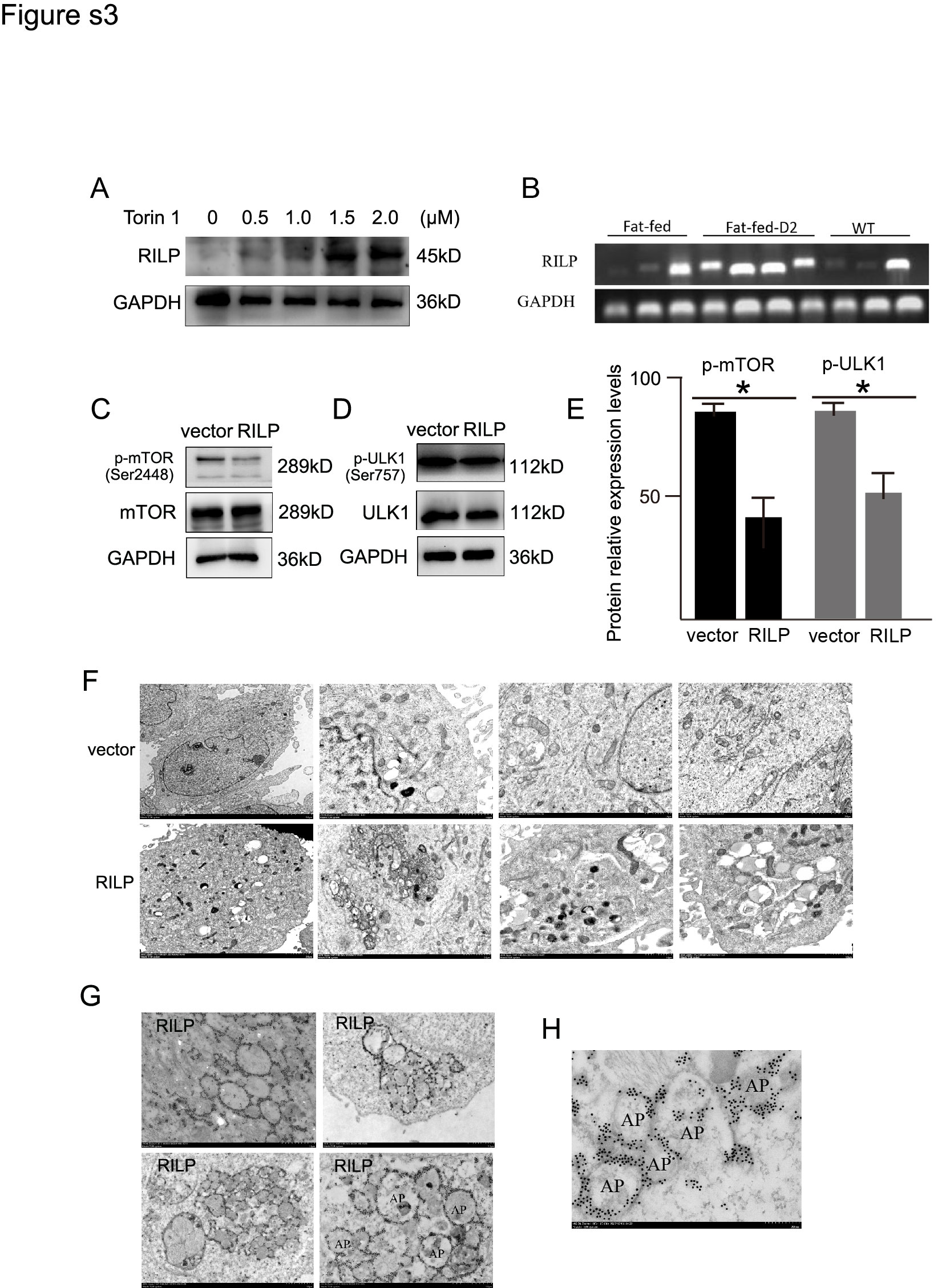

Supplement: Supplementary file 1 [file cells-13-01313-s001.zip › Figure_s3.jpg]

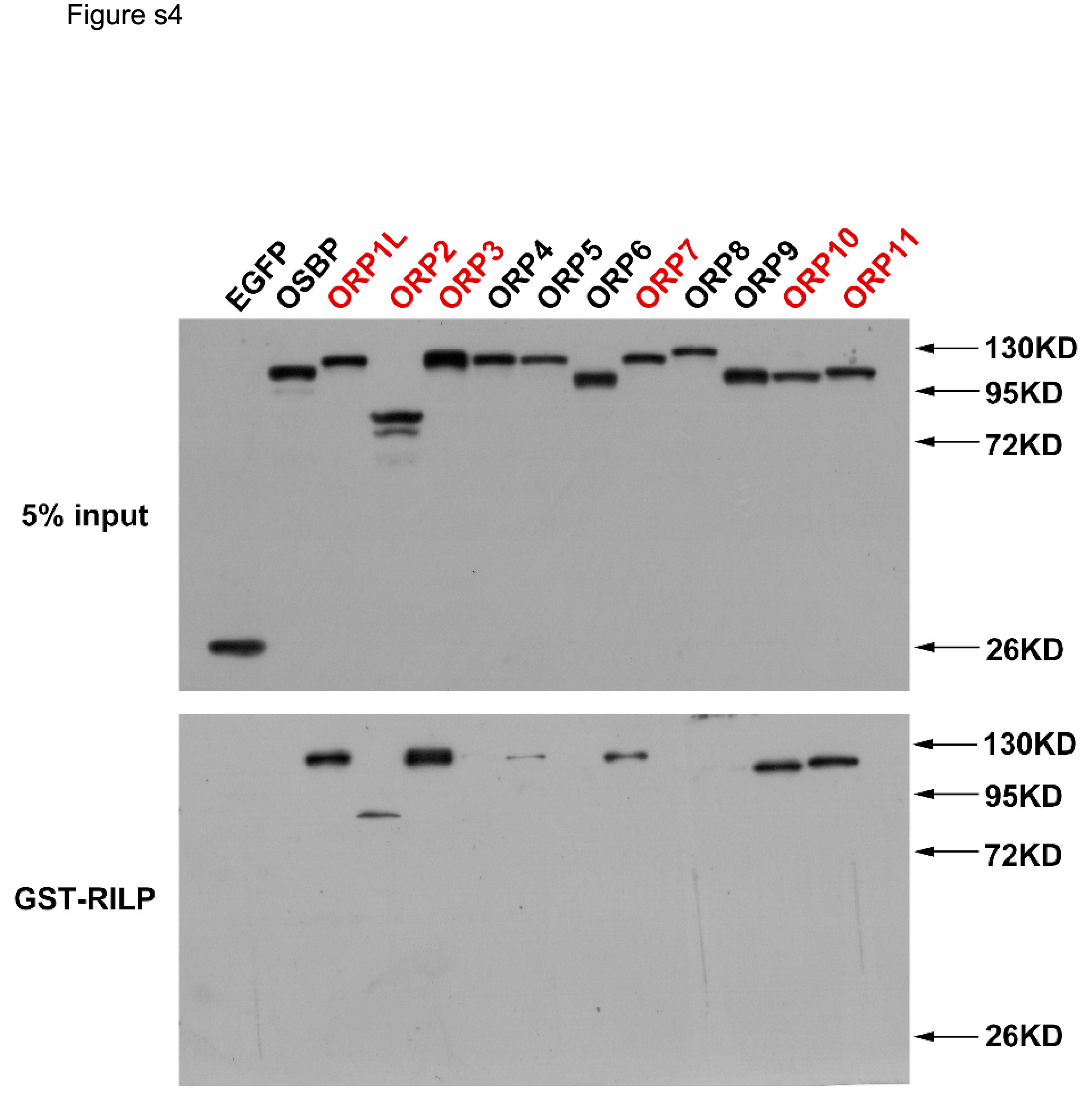

Supplement: Supplementary file 1 [file cells-13-01313-s001.zip › Figure_s4.jpg]
